# Lifetime Prevalence of Nonspecific Low Back Pain in Adolescents: A Cross-sectional Epidemiologic Survey

**DOI:** 10.1097/PHM.0000000000001720

**Published:** 2021-02-19

**Authors:** Stefano Masiero, Fabio Sarto, Manuela Cattelan, Diego Sarto, Alessandra Del Felice, Francesco Agostini, Anna Scanu

**Affiliations:** From the Department of Neuroscience, Section of Rehabilitation (SM, ADF), Padova Neuroscience Center (SM, ADF), School of Human Movement Science (FS, DS), and Department of Statistical Sciences (MC), University of Padova, Padova; Department of Anatomical and Histological Sciences, Legal Medicine and Orthopedics, Sapienza University of Rome, Rome (FA); and Rheumatology Unit, Department of Medicine-DIMED, University of Padova, Padova, Italy (AS).

**Keywords:** Nonspecific Low Back Pain, Teenagers, Sports, Electronic Devices, Disabling Low Back Pain, Healthcare Professional

## Abstract

Many nonconclusive studies have been conducted on low back pain (LBP) in adolescents and associated factors.

**Objective:**

The aim was to assess the lifetime prevalence and associated factors of LBP in adolescents.

**Materials and Methods:**

A questionnaire was administered in high school students (14–19-yr-old participants) in Veneto region (Italy). The self-administered, structured questionnaire included anthropometric data; psychologic factors and lifestyle; presence, intensity, and family history of LBP; referral to professional health care for LBP; and a short version of the International Physical Activity Questionnaire.

**Results:**

A total of 6281 adolescents were recruited; 5204 questionnaires were included in the final analysis. A total of 2549 (48.98%) students reported one or more LBP episodes and 723 (13.89%) reported nonspecific disabling lumbar pain (i.e., no underlying pathology); 1040 (41.11%) subjects with LBP consulted a healthcare professional. A significant association emerged for LBP with sex (female), positive family history, time spent sitting or using electronic devices, sleep deprivation (<5 hrs/night), and low level of physical activity.

**Conclusion:**

In a large sample of adolescents, LBP lifetime prevalence is high and often associated with disabling pain and sedentary lifestyle, requiring professional care. These findings may support the development of prevention and treatment strategies of LBP in adolescents, reducing the risk of developing chronic pain.

## Abstract 2

Supplemental digital content is available in the text.

**What Is Known**

- Nonspecific LBP is relatively common among adolescents, mostly in females, and is associated with age, positive family history, lifestyle, and sleep deprivation. Sport practice lowers the incidence of LBP.

**What Is New**

- LBP is often associated with disabling pain and requires professional intervention. Identifying adolescent LBP and associated factors may reduce the risk of chronic pain.

Nonspecific low back pain (LBP) is defined as pain and discomfort localized between the costal margin and the lower gluteus folds, with or without radiation to the lower limbs, not attributed to specific and/or known diseases.^1,2^

The diagnosis of this condition in adolescence is of exclusion, for example, in the absence of infections, tumors, spondylolysis, spondylolisthesis, juvenile osteochondrosis of the spine (Scheuermann disease), and rheumatic diseases.^3^ This aspect must be more emphasized in adult patients (age >20 yrs), owing to the lower frequency of nonspecific LBP in this age group.^4^

Epidemiologic data show that most LBP cases in adolescents are nonspecific. A recent systematic review indicates that the lifetime prevalence of nonspecific LBP in children and adolescents varies between 11.60% and 83.56%.^5^ This wide range is likely because of the heterogeneity and the different cultural and social norms of individuals included.^6–10^

Indeed, no consensus exists on a sex difference in LBP prevalence,^6,11,12^ whereas data on the association of height, weight, body mass index (BMI), and anthropometric factors are still inconclusive.^13,14^

Among the factors associated with the onset, progression, and outcome of this condition, lifestyle factors such as smoking, hours of sleep per night, or long hours sitting (computer, school) and psychosocial factors such as depression, stress, poor academic performance, and perceived weight of backpacks are reported.^9,15–17^

Physical inactivity is supposed to be associated with higher risk for recurrent LBP, but there are contradictory results reported regarding the association of LBP with physical activity and physical fitness level.^18,19^ On this ground, the link between LBP and physical activity has been described as a U-shaped relationship, where increased risk was found for both subjects with a sedentary lifestyle and those practicing strenuous activities.^20^

Previous studies reported the prevalence of LBP and associated risk factors in 7542 teenagers aged 13–15 yrs, with a definition of prevalence as the presence of LBP over a 1-yr period.^21,22^ In this study’s cohort, 20.5% of teenagers reported one or more episodes of LBP. Nine hundred (76.3%) had consulted a healthcare professional; a significant association with sex (female), family history, and physical inactivity emerged, whereas anthropometrics or lifestyle items did not correlate.

Nonspecific LBP in adolescents, associated with the risk of developing *chronic* pain,^8,15^ has a high impact on the individual as well as on society, with important economic consequences. Therefore, research to highlight LBP causes and develop preventive measures is of utmost importance.

The aim of this study was to investigate the lifetime prevalence and associated factors of nonspecific adolescent LBP to improve knowledge on causative factors and allow measures to prevent chronicity. The aim was to determine the impact of perceived pain on the daily lives and activities of adolescents.

## MATERIALS AND METHODS

### Participants and Study Design

This is a cross-sectional epidemiologic survey, conducted between February and May 2018 (2017–2018 academic year). Inclusion criteria were students, residing both in urban and rural areas, between the age of 14 and 19 yrs attending high schools in Veneto Region (Italy) who agreed to participate in the survey. Schools were selected on the basis of their zip codes (odd numbers included). Exclusion criteria were already diagnosed spinal pathologies that might cause LBP (Scheuermann disease, spondylolysis, spondylolisthesis, facet arthropathy, sacroiliac joint pain, spondylitic stenosis, compression fracture, and rheumatic diseases) or previous back surgery and back pain areas different from the lumbar region.

### Questionnaire and Data Collection

The study was based on a structured, self-administered questionnaire, ad hoc designed for this epidemiologic survey, consisting of multiple-choice questions.^21^ Students completed the questionnaires using a laptop, a tablet, or a smartphone. On the day of data collection, the questionnaires were presented by a member of the research team to students in each class during teaching hours: items were illustrated and the students were explained how to fill it in. A temporary password was provided for each class to access the online questionnaire. The questionnaire was anonymous. A pilot study on 78 schools was conducted to test the questionnaire for ease of access, nonequivocal items, and time needed to fill it out. The time required to complete it was on average 20 mins.

The first section consisted of questions regarding demographic items (age, height, weight, BMI, sex). A short version of the International Physical Activity Questionnaire (IPAQ-SF) was included in this section to measure physical activity levels.^23^ This seven-item questionnaire was developed as a tool for monitoring physical activity and inactivity over the last 7 days. It is divided into four categories: vigorous intensity, moderate intensity, walking, and sitting. For each of these categories, students had to declare for how many days and how many minutes they spent in a specific category of activity. Four subscores expressed in metabolic equivalent of task–minutes per week were obtained by multiplying these data by the intensity coefficients, according to the IPAQ protocol (ipaq.ki.se). Furthermore, a total score was calculated by adding the three subscores related to vigorous and moderate-intensity activity and walking.

According to IPAQ guidelines, individuals who did not answer to the minutes of daily activity or reported more than 960 mins of daily activity were discarded.

The second section collected information regarding type of sporting activity (soccer, volleyball, basketball, athletics, swimming, fitness, rugby, other) and frequency of training sessions (number of weekly hours). Other items investigated lifestyle, such as the daily number of hours of sleep and daily hours with electronic devices (laptop, tablet, or smartphone).

This section ended with items investigating the presence of LBP (at least one episode of LBP in their life), that is, any nonoccasional pain that in some way limited the student in daily activities. The definition of nonspecific LBP followed the European Guidelines for prevention of LBP^1^: nonspecific LBP is pain and discomfort localized below the costal margin and above the inferior gluteal folds, with or without leg pain, with no other associated back pathology. The final section consisted of questions on the maximum and average level of perceived pain (measured with a numerical rating scale, 0 = no pain, 10 = worst pain) and the need of medical examination. In addition, students were asked whether they ever had to give up social activities because of LBP; those who did were assigned to the disabling LBP group (Dis).

### Ethical Issues

The study was conducted in accordance with the Declaration of Helsinki, and the protocol was approved by the Ethics Committee of the University Hospital of Padova (n. HEC-DSB/02-19).

Legal guardians signed an informed consent. All procedures were performed according to the Declaration of Helsinki. The STROBE cross-sectional checklist was used for reporting (see Supplemental Checklist, Supplemental Digital Content 1, http://links.lww.com/PHM/B243).^24^

### Study Size

This study was developed with an explorative aim, with no previous hypothesis about the prevalence of LBP in the population. Thus, computation of the sample size was not performed. However, the number of questionnaires completed ensures a high statistical power for each association test performed, all above 90%.

### Statistical Analysis

Descriptive statistics are reported in terms of absolute values and percentages. Univariate analyses on the association between the presence of pain and other categorical variables were performed using chi-square tests. A multivariate analysis, which allows simultaneous evaluation of the association of the different variables with pain, was performed through logistic regression. Evaluation of the significance of covariates in the logistic model was based on the likelihood ratio statistic. In case of missing data, the analyses were performed on individuals with complete answers. Association between the type of lower back pain and the other variables was assessed using the chi-square test. Analyses were run using the statistical software R.^25^ Statistical significance was set at *P* < 0.05.

## RESULTS

Twenty-four schools participated in this survey; 6281 questionnaires were completed, and response rate was 100%. Supplementary Figure 1 (Supplemental Digital Content 2, http://links.lww.com/PHM/B244) summarizes the inclusion process. Participants had an average age of 16.93 ± 1.92 yrs (range, 14–19 yrs). Incomplete questionnaires or those with data in the category of exclusion criteria (*n* = 300, 4.78%) were not included. The resulting data set consisted of 5981 observations (3709 [62%] female students). Supplementary Figure 2 (Supplemental Digital Content 3, http://links.lww.com/PHM/B245) shows the distribution according to age and sex.

Fifty-five percent (55.6%, *n* = 3326) of students reported having suffered from back pain (Table 1). These were then divided according to the area of pain: neck pain or LBP. A total of 729 students were excluded because they suffered from neck pain, whereas 48 students were further excluded because they reported having suffered from back pain but did not specify the area; final analyses were based on 5204 responses. There were 2549 (48.98%) participants who reported one or more LBP episodes. The test on the association between sex and back pain distribution showed a significant association (*P* < 0.001), with female students suffering more than male students (Table 1). No significant association between BMI and LBP (*P* = 0.63) emerged.

**TABLE 1:** Distribution of back pain and areas of back pain by sex

Variable		Total	F, *n*	M, *n*	F, %	M, %	*P*
Pain	No	2655 (44.39%)	1469	1186	55.33	44.67	<0.001
	Yes	3326 (55.61%)	2240	1086	67.35	32.65	
Area							
	No pain	2655	1469	1186	55.33	44.67	<0.001
	NP	729	546	183	74.90	25.10	
	LBP	2549	1673	876	65.63	34.37	

*P* is the *P* value of the test of association.

F indicates female; M, male; NP, neck pain.

Table 2 shows that LBP frequency was higher in students who did not practice sports regularly (51.83%) (*P* < 0.001). There was no significant association between IPAQ scores and back pain scores (*P* = 0.73) (Table 2).

**TABLE 2:** Regularly playing sports and low back pain

	No Pain, *n*	LBP, *n*	No Pain, %	LBP, %	*P*
Sport					
No	737	793	48.17	51.83	<0.001
Yes	1789	1642	52.14	47.86	
NA	129	114	53.09	46.91	
Sport played					
None/NA	976	1031	48.63	51.37	
Other	551	518	51.54	48.46	
Athletics	69	78	46.94	53.06	
Basketball	127	91	58.26	41.74	
Soccer	282	210	57.32	42.68	
Swimming	159	119	57.19	42.81	
Body building	306	271	53.03	46.97	
Volleyball	160	212	43.01	56.99	
Rugby	25	19	56.82	43.18	
MET levels					
Low	440	429	43.78	42.69	0.730
Moderate	851	791	45.12	41.94	
High	1364	1329	44.84	43.69	

%: Distribution percentage.

NA indicates not available; MET, metabolic equivalent of task.

It was observed that the percentage of students with LBP decreased with hours of sleep (*P* < 0.001), whereas increased with the number of hours spent sitting (*P* < 0.001), time spent using electronic devices (*P* < 0.001), and family history (*P* < 0.001) (Table 3).

**TABLE 3:** Hours of sleep, sitting, spent using tablets/PC/phones, family history and back pain

	No Pain, *n*	LBP, *n*	No Pain, %	LBP, %	*P*
Hours of sleep					
<5	72	121	37.31	62.69	<0.001
5–7	1155	1201	49.02	50.98	
7–9	1357	1175	53.59	46.41	
>9	71	52	57.72	42.28	
Hours sitting					
<5	165	142	53.75	46.25	<0.001
5–8	1777	1601	52.61	47.39	
>8	713	806	46.94	53.06	
Hours spent using tablets/PC/phones					
<2	463	395	53.96	46.04	<0.001
2–5	1515	1379	52.35	47.65	
5–7	471	529	47.10	52.90	
>7	206	246	45.58	54.42	
Family history					
No	1484	971	60.45	39.55	<0.001
Yes	1171	1578	42.60	57.40	

*P* is the *P* value of the test of association.

Multivariate analysis on the association of different variables with LBP showed a significant effect of sex, age, sport, hours of sleep, and family history. Given the other covariates, the ratio of probabilities of having had LBP and not having had it for a male student is 0.693 times the same ratio for female students (*P* < 0.001). Students who practice sports were less likely to suffer from back pain (*P* = 0.002). Students who sleep more than 5 hrs per night had a lower chance of reporting LBP (*P* = 0.008). Lastly, the ratio of probabilities of having LBP and not having it for students with positive family history was 1.87 times the same ratio for those without family history (*P* < 0.001).

A total of 1048 (41.11%) students sought medical advice (714 female students), of whom 399 had a disabling LBP. Of the 2549 subjects reporting LBP, 723 (28.36%) had a disabling LBP (Table 4).

**TABLE 4:** Distribution of type of pain and sex or hours of sleep

	Non-Dis, *n*	Dis, *n*	Non-Dis, %	Dis, %	*P*
Sex					
F	1108	491	64.95	67.91	0.170
M	598	232	35.05	32.09	
Hours of sleep					
<5	64	50	56.14	43.86	<0.001
5–7	801	342	70.08	29.92	
7–9	816	309	72.53	27.47	
>9	25	22	53.19	46.81	

*P* is the *P* value of the test of association.

F indicates female; M, male; Dis, disabling LBP.

The only significant association with disabling LBP was hours of sleep less than 5 hrs or more than 9 hrs per night (*P* < 0.001) (Table 4).

**FIGURE 1:**
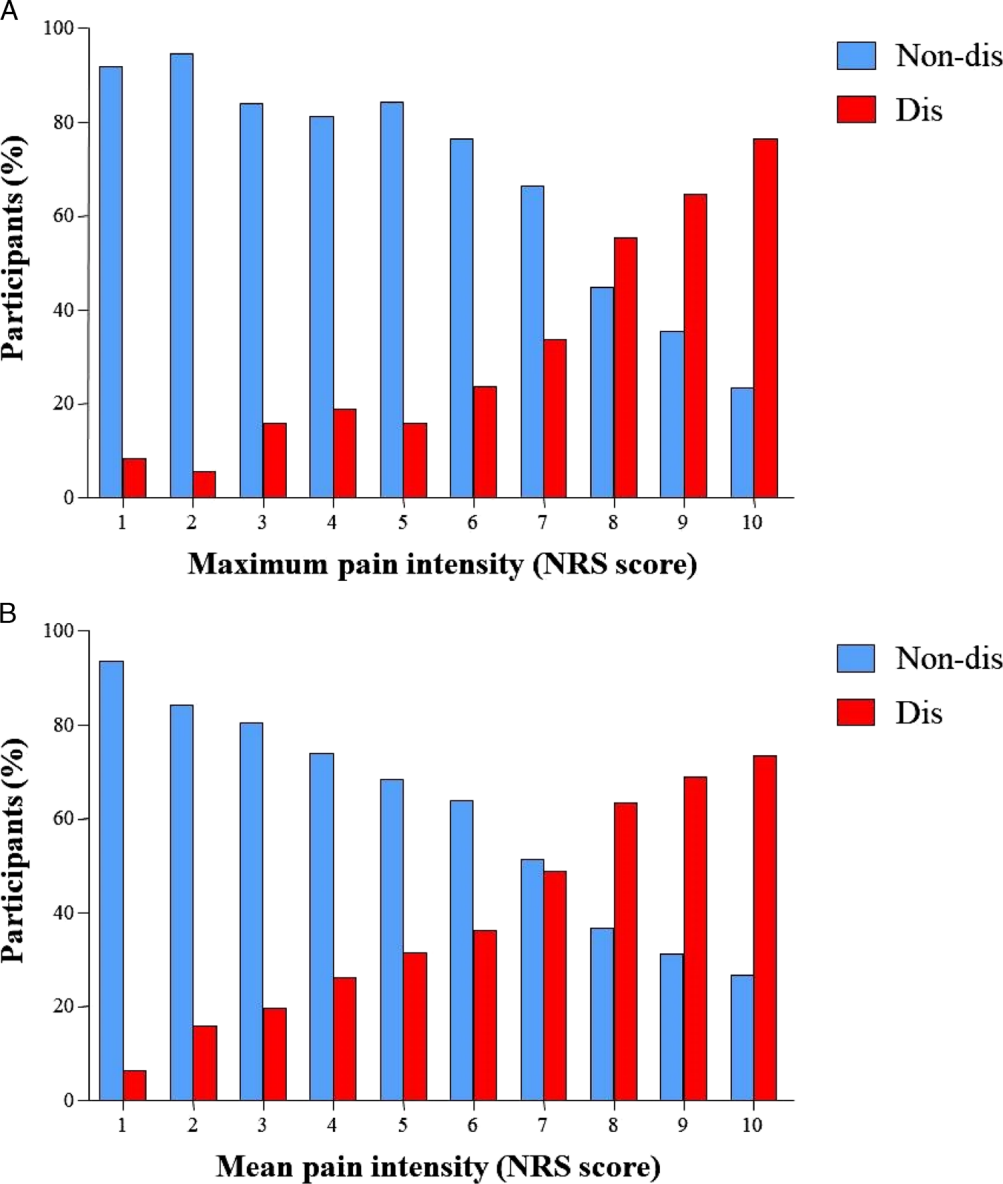
Distribution of disabling (dis) LBP or non-dis LBP and (A) maximum pain intensity and (B) mean pain intensity.

The distribution of maximum pain intensity showed higher numerical rating scale scores for students suffering from a disabling LBP than those suffering from nondisabling LBP (*P* < 0.001) (Fig. 1A). A significant association was also present between disabling LBP and the mean pain intensity (*P* < 0.001) (Fig. 1B).

## DISCUSSION

This study demonstrated a high lifetime prevalence of nonspecific LBP and associated factors in adolescents in Veneto Region (Italy). In addition, the lifetime prevalence of nonspecific disabling LBP in adolescents is reported, that is, which limited and/or hampered daily life activities and requested medical consultation.

There is general agreement that LBP in adolescents is a health problem requiring much more attention and resources than those devoted at the moment of this writing. In light of lifestyle changes in new generations, studies analyzing LBP risk factors are crucial. The results can be used in the preventive or educational field, which today represents one of the most effective therapeutic approaches in LBP treatment to avoid pain chronicity and the subsequent economic consequences.^8,15^

The results support the evidence that nonspecific LBP is common in adolescence.^5^ Indeed, 55.61% (3326 subjects) of students reported having suffered from back pain at least once in their life and 42.62% (2549 subjects) reported one or more episodes of LBP.

LBP lifetime prevalence is a suggestive measure in adolescents: they are more likely to remember pain episodes that occurred also many years earlier, probably because of their emotional, psychologic, and relational life impact.^26^

In agreement with previous studies, the highest prevalence of LBP was found in the female sex, probably because of a different pain threshold and pain symptom perception.^10,27^ Other possible related factors are the greater flexibility of the spine compared with males and the possible changes induced by hormonal changes on the appearance and perception of pain.^17,28^

An association between LBP and BMI was not observed. This is in agreement with previous reports suggesting that nonspecific LBP in adolescents is more related to an incorrect lifestyle.^5,8,21^ Conversely, it has been demonstrated that in adults, the risk of LBP increases in parallel with BMI and may be modulated by physical activity.^29^

A clear-cut relationship between physical activity levels (investigated through IPAQ) and LBP was not identified; in fact, it emerged that students who regularly practice sports (at least 2–3 hrs a week) were less likely to suffer of LBP. These data confirm that physical activity, improving muscle elasticity, strength, and likely increasing pain threshold,^30^ can prevent the onset of LBP.^31^

The relationship between physical activity levels and LBP is controversial and widely discussed. In fact, it has been observed that both an insufficient as well as an excessive motor activity predisposes to the development of LBP with a U-shaped relationship.^32^ Specific skills required in different sports expose the vertebral discs to considerable pressures; sports in general increase the risk of injuries, which may lead to LBP.^33^

An association between LBP and hours of sleep was found. It was observed that sleeping more than 5 hrs a night was associated with a lower probability of suffering from LBP, suggesting that sleep may be a protective factor.

An association between LBP and use of electronic devices (laptop, tablet, or a smartphone) and hours spent sitting was also found,^34^ suggesting that the two factors may be related. An inadequate prolonged static posture, adopted using these devices, might generate musculoskeletal overload, activating pain receptors.^34^

The results of this study confirm a predisposition to LBP in subjects with positive family history, likely owing to the genetic factors involved.^21,22,28,35^ However, the family environment may also play a role because it has been observed that parents may impact on pain threshold level and symptoms complain, heightening the prevalence of disabling LBP in this subsample.^21,22,28,35^ Apprehension and anxiety by one or both parents can prompt health-seeking behavior, especially when pain presents a chronic course.^36^

Of 2549 students with LBP, 723 (28.36%) reported at least one episode of nonspecific disabling LBP. However, age and sex did not seem to influence the type of LBP (disabling or not).

An association between disabling LBP and hours of sleep was found. Although sleep may be a protective factor for LBP, it has also been reported sufferers of disabling LBP have a poor sleep quality, negatively affecting both the perception of pain and the quality of life.^37^

The presence of a disabling pain also increased seeking for healthcare consultation (55.19%). These data are in contrast with previous studies reporting that only 2%–15% of children and adolescents with episodes of LBP require a medical and/or instrumental evaluation.^11,28^ This difference could be a result of the definition of LBP that was used. Most included studies used structured or semistructured questionnaires with only a partial definition of nonspecific LBP; hence, it could be misleading to draw comparisons with other studies. In fact, according to the definition in this study, the LBP was nonspecific and had to limit the adolescent’s daily activities.

Of note, this study recruited more than 5000 adolescents, whereas surveys in this area of research usually consider less than 1500 participants, and most of these do not reach 500 individuals.^5^ The analysis of such a large sample allows valid and reliable results.

To sum up, these findings stress the need to focus therapeutic efforts with adequate prevention and education programs targeted both to adolescent and relevant adults (e.g., parents, teachers, sports trainers).

### Limitations

This study has some limitations. The main one is the use of an ad hoc questionnaire, which allowed obtaining data from a large sample but is difficult to compare with other studies.

Because different types of schools were included, some minimal bias may be introduced in the study, such as the request for longer autonomous hours in equivalent of grammar schools—thus more time spent sitting rather than exercising. Another limitation is that, despite the fact that adolescents can suffer from pain in different segments of the spine, even simultaneously, the formulation of the questionnaire in this study allowed investigating only one location. Furthermore, the impact of passive and active smoking was not considered. Data on the number and the duration of pain episodes were not included. An investigation of such issues is underway.

Another aspect that was not considered is the perception of the weight of backpacks by adolescents. It has been reported that, rather than the actual and objective weight of the backpack, it is the student’s perception of weight that is associated with LBP.^17^

Another limitation may have been the imperfect recall of events, which is inherently related to this methodologic approach and cannot be otherwise corrected.

A potential inclusion bias may have been introduced by the exclusion of subjects with a known diagnosis underlying back pain: although this subgroup does experience LBP, the focus of the present study was LBP not related to spinal diseases. The authors reckon that a small sample of individuals in which a diagnosis was not already made might have been included, but they are confident that this may not have substantially impacted on final results.

Another limitation to be considered is the incomplete population sample: for convenience, schools were included on an alternating basis (odd zip codes). Although the randomization method is robust, it needs to be acknowledged that not the whole population aged 14–19 yrs was included.

Lastly, other psychosocial aspects such as depression, anxiety, distress, and exposure to stressful life events were not considered.

## CONCLUSION

The results of this study support the evidence that nonspecific LBP is relatively common among adolescents (mostly in females), especially if they are sedentary and heavy users of electronic devices. A positive family history of LBP is associated with disabling LBP, and family environment (apprehension/anxiety/coping skills) might also play a substantial role. Sleeping more than 5 hrs a night is associated with a lower probability of having LBP. Frequently, adolescents with LBP, particularly those with disabling one, consult a healthcare professional. Practicing sport regularly seems to be associated with a lower probability of having LBP. Further studies are needed to identify those at risk and to define more clearly the role of sports activities in this age group, to promote prevention interventions and plan a personalized rehabilitation program.

## Supplementary Material
