# Supplementary material for: Lifetime Prevalence of Nonspecific Low Back Pain in Adolescents: A Cross-sectional Epidemiologic Survey
Source: Am J Phys Med Rehabil. 2021 Feb 19;100(12):1170–5. doi: 10.1097/PHM.0000000000001720 (PMC9988216; doi:10.1097/PHM.0000000000001720)
Supplement: Supplementary file 1 [file ajpmr-100-1170-s001.docx]

**Lifetime prevalence of non-specific low back pain in adolescents: a cross-sectional epidemiological survey**

Stefano Masieroa,b, Fabio Sartoc, Manuela Catteland, Diego Sartoc, Alessandra Del Felicea,b,

Francesco Agostinie, Anna Scanuf

aDepartment of Neuroscience, Section of Rehabilitation, University of Padova, Italy.

bPadova Neuroscience Center, University of Padova, Italy.

cSchool of Human Movement Science, University of Padova, Italy.

dDepartment of Statistical Sciences, University of Padova, Italy.

eDepartment of Anatomical and Histological Sciences, Legal Medicine and Orthopedics, Sapienza University

of Rome, Italy.

fRheumatology Unit, Department of Medicine-DIMED, University of Padova, Italy.

**Corresponding author**

Alessandra Del Felice, MD, PhD

Department of Neuroscience, Section of Rehabilitation

University of Padova, Padova, Italy

Via Giustiani, 3, 35128 Padova

Tel: +39-(0)49-8211270

e-mail: alessandra.delfelice@unipd.it

**Abstract word count:** 234

**Main text word count:** 3054

**Number of tables/figures:** 5

**Number of references**: 37

No funding was received for this study

**ABSTRACT**

**Background:** Many non-conclusive studies have been conducted on low back pain (LBP) in adolescents and associated factors.

**Objective:** The aim was to assess lifetime prevalence and associated factors of LBP in adolescents.

**Materials and methods:** A questionnaire was administered in high-schools (14-19 years old participants) in Veneto region (Italy). The self-administered, structured questionnaire included anthropometric data, psychological factors and lifestyle, presence, intensity and family history of LBP, referral to professional healthcare for LBP, and a short version of the International Physical Activity Questionnaire.

**Results:** Six-thousand-two-hundred-eighty-one adolescents were recruited; 5204 questionnaires were included in final analysis. Two-thousand-five-hundred-forty-nine (48.98%) students reported one or more LBP episodes and 723 (13.89%) non-specific disabling lumbar pain (i.e. no underlying pathology). Thousand and forty (41.11%) subjects with LBP consulted a healthcare professional. A significant association emerged for LBP with sex (female), positive family history, time spent sitting or using electronic devices, sleep deprivation (less than five hours/night) and low-level of physical activity.

**Conclusion:** In a large sample of adolescents, LBP lifetime prevalence is high and often associated with disabling pain and sedentary lifestyle, requiring professional care. These findings may support the development of prevention and treatment strategies of LBP in adolescents, reducing the risk of developing chronic pain.

**Keywords:** Non-specific low back pain; teenagers; sports; electronic devices; disabling low back pain; healthcare professional.

**Summary Text**

**What is Known:** Non-specific LBP is relatively common among adolescents, mostly in females, and is associated with age, positive family history, lifestyle, and sleep deprivation. Sport practice lowers the incidence of LPB.

**What is new:** LBP is often associated with disabling pain and requires professional intervention. Identifying adolescent LPB and associated factors may reduce the risk of chronic pain.

**INTRODUCTION**

Non-specific low back pain (LBP) is defined as pain and discomfort, localized between the costal margin and the lower gluteus folds, with or without radiation to the lower limbs, not attributed to specific and/or known diseases^1, 2^.

The diagnosis of this condition in adolescence is of exclusion, e.g. in the absence of infections, tumors, spondylolysis, spondylolisthesis, juvenile osteochondrosis of the spine (Scheuermann's disease), and rheumatic diseases^3^. This aspect must be more emphasized in adult patients (age> 20 years), due to the lower frequency of non-specific LBP in this age group^4^.

Epidemiological data show that the majority of LBP cases in adolescents are non-specific. A recent systematic review indicates that the lifetime prevalence of non-specific LBP in children and adolescents varies between 11.60% and 83.56%^5^. This wide range is likely due to the heterogeneity and to the different cultural and social norms of individuals included^6-10^.

Indeed, no consensus exists on a gender difference in LBP prevalence^6, 11, 12^ while data on the association of height, weight, body mass index (BMI), and anthropometric factors are still inconclusive^13, 14^.

Among the factors associated with the onset, progression and outcome of this condition, lifestyle such as smoking, hours of sleep per night, or long hours sitting (computer, school) and psychosocial factors such as depression, stress, poor academic performance, and perceived weight of backpacks, are reported^9, 15-17^.

Physical inactivity is supposed to be associated with higher risk for recurrent LBP, but there are contradictory results reported regarding the association of LBP with the physical activity and physical fitness level^18, 19^. On this ground, the link between LBP and physical activity has been described as a U-shaped relationship, where increased risk was found for both subjects with a sedentary lifestyle and those practicing strenuous activities^20^.

Previous studies reported the prevalence of LBP and associated risk factors in 7542 teenagers aged 13-15 years, with a definition of prevalence as the presence of LBP over a 1 year period^21,22^. In our cohort, 20.5% of teenagers reported one or more episodes of LBP. Ninety-hundred (76.3%) had consulted a healthcare professional; a significant association with gender (female), family history and physical inactivity emerged, whereas anthropometrics or lifestyle items did not correlate.

Non-specific LBP in adolescents, associated with the risk of developing chronic pain^8,15^, has a high impact on the individual as well as on society, with important economic consequences. Therefore, research to highlight LBP causes and develop preventive measures is of utmost importance.

The aim of this study was to investigate the lifetime prevalence and associated factors of non-specific adolescent LPB to improve knowledge on causative factors and allow measures to prevent chronicity. We aimed at determining the impact of perceived pain on the daily lives and activities of adolescents.

**MATERIALS AND METHODS**

*Participants and study design*

This is a cross-sectional epidemiological survey, conducted between February and May 2018 (2017-2018 academic year). Inclusion criteria were: students, residing both in urban and rural areas, between the age of 14 and 19 attending high schools in Veneto Region (Italy) who agreed to participate in the survey. Schools were selected on the basis of their zip-codes (odd numbers included). Exclusion criteria were: already diagnosed spinal pathologies that might cause LBP (Scheuermann’s disease, spondylolysis, spondylolisthesis, facet arthropathy, sacroiliac joint pain, spondylitic stenosis, compression fracture, and rheumatic diseases) or previous back surgery; back pain areas different from the lumbar region.

*Questionnaire and data collection*

The study was based on a structured, self-administered questionnaire, *ad hoc* designed for this epidemiological survey, consisting of multiple-choice questions^21^. Students completed the questionnaires using a laptop, a tablet or a smartphone. On the day of data collection, the questionnaires were presented by a member of the research team to students in each class during teaching hours: items were illustrated and the students were explained how to fill it in. A temporary password was provided for each class to access the online questionnaire. The questionnaire was anonymous. A pilot study on 78 schools was conducted to test the questionnaire for ease of access, non-equivocal items, time needed to fill it. The time required to complete it was on average 20 minutes.

The first section consisted of questions regarding demographic items (age, height, weight, body mass index, sex). A short version of the International Physical Activity Questionnaire (IPAQ-SF) was included in this section to measure physical activity levels^23^. This 7-item questionnaire was developed as a tool for monitoring physical activity and inactivity over the last seven days. It is divided into four categories: vigorous intensity, moderate intensity, walking and sitting. For each of these categories, students had to declare for how many days and how many minutes they spent in a specific category of activity. Four sub-scores expressed in Metabolic Equivalent of Task (MET)-min/week were obtained by multiplying this data by the intensity coefficients, according to the IPAQ protocol (ipaq.ki.se). Furthermore, a total score was calculated by adding the three sub-scores related to vigorous and moderate intensity activity and walking.

According to IPAQ guidelines, individuals who did not answer to the minutes of daily activity or reported more than 960 minutes of daily activity were discarded.

The second section collected information regarding type of sporting activity (soccer, volleyball, basketball, athletics, swimming, fitness, rugby, other) and frequency of training sessions (number of weekly hours). Other items investigated lifestyle, such as the daily number of hours of sleep and daily hours with electronic devices (laptop, tablet, or smartphone).

This section ended with items investigating the presence of LBP (at least one episode of LBP in their life), i.e. any non-occasional pain that in some way limited the student in daily activities. The definition of non-specific LBP followed the European Guidelines for prevention of LBP^1^: non-specific LPB is a pain and discomfort localized below the costal margin and above the inferior gluteal folds, with or without leg pain with no other associated back pathology. The final section consisted of questions on the maximum and average level of perceived pain (measured with a Numerical Rating Scale - NRS, 0 = no pain, 10 = worst pain), and the need of medical examination. In addition, students were asked if they ever had to give up social activities due to LBP; those who did were assigned to the disabling LBP group (Dis).

*Ethical issues*

The study was conducted in accordance with the Declaration of Helsinki, and the protocol was approved by the Ethics Committee of the University Hospital of Padova (n. HEC-DSB/02-19).

Legal guardians signed an informed consent. All procedures were performed according to the Declaration of Helsinki. The STROBE cross sectional check list was used for reporting (See Supplemental Checklist, Supplemental Digital Content 1, http://links.lww.com/PHM/B243)^24^.

*Study size*

This study was developed with an explorative aim, with no prior hypothesis about the prevalence of LBP in the population. Thus, computation of the sample size was not performed. However, the number of questionnaires completed ensures a high statistical power for each association test performed, all above 90%.

*Statistical analysis*

Descriptive statistics are reported in terms of absolute values and percentages. Univariate analyses on the association between the presence of pain and other categorical variables were performed using chi-square tests. A multivariate analysis, which allows to evaluate simultaneously the association of the different variables with pain, was performed through logistic regression. Evaluation of the significance of covariates in the logistic model was based on the likelihood ratio statistic. In case of missing data, the analyses were performed on individuals with complete answers. Association between the type of lower back pain and the other variables was assessed using the chi-square test. Analyses were run using the statistical software R^25^. Statistical significance was set at p<0.05.

**RESULTS**

Twenty-four schools participated in this survey; 6281 questionnaires were completed; response rate was 100%. Supplementary Figure 1 (Supplemental Digital Content 2, http://links.lww.com/PHM/B244) summarizes the inclusion process. Participants had an average age of 16,93±1,92 (range 14-19). Incomplete questionnaires or those with data in the category of exclusion criteria (n=300, 4.78%) were not included. The resulting data set consisted of 5981 observations [3709 (62%) females]. Supplementary Figure 2 (Supplemental Digital Content 3, http://links.lww.com/PHM/B245) shows the distribution according to age and sex.

Fifty-five percent (55.6%, n. 3326) of students reported having suffered from back pain (Table1). These were then divided according to the area of pain: neck pain (NP) or LBP. Seven hundred twenty-nine were excluded because they suffered from neck pain, while 48 students were further excluded because they reported having suffered from back pain but did not specify the area; final analyses were based on 5204 responses. Two thousand five hundred forty-nine (48.98%) reported one or more LBP episodes. The test on the association between sex and back pain distribution showed a significant association (p<0.001) with females suffering more than males (Table1). No significant association between BMI and LBP (p=0.63) emerged.

Table 2 shows that LBP frequency was higher in students who did not practice sports regularly (51.83%) (p<0.001). There was no significant association between IPAQ questionnaire scores and back pain scores (p = 0.73) (Table 2).

We observed that the percentage of students with LBP decreased with hours of sleep (p<0.001), while increased with the number of hours spent sitting (p<0.001), time spent using electronic devices (p<0.001) and family history (p<0.001) (Table 3).

Multivariate analysis on the association of different variables with LBP showed a significant effect of sex, age, sport, hours of sleep, and family history. Given the other covariates, the ratio of probabilities of having had LBP and not having had it for a male is 0.693 times the same ratio for females (p<0.001). Students who practice sports were less likely to suffer from back pain (p=0.002). Students who sleep more than five hours per night had a lower chance of reporting LBP (p=0.008). Lastly, the ratio of probabilities of having LBP and not having it for students with positive family history was 1.87 times the same ratio for those without family history (p<0.001).

One thousand forty-eight (41.11%) students sought medical advice (714 females), of whom 399 had a disabling LBP. Of the 2549 subjects reporting LBP, 723 (28.36%) had a disabling LBP (Table 4).

The only significant association with disabling LBP was hours of sleep less than five or more than nine hours per night (p<0.001) (Table 4).

The distribution of maximum pain intensity showed higher NRS scores for students suffering from a disabling LBP than those suffering from non-disabling LBP (p<0.001) (Figure 1A). A significant association was also present between disabling LBP and the mean pain intensity (p<0.001) (Figure 1B).

**DISCUSSION**

This study demonstrated a high lifetime prevalence of non-specific LBP and associated factors in adolescents in Veneto Region (Italy). In addition, we report the lifetime prevalence of non-specific disabling LPB in adolescents, i.e. that limited and/or hampered daily life activities and requested medical consultation.

There is general agreement that LBP in adolescents is a health problem requiring much more attention and resources than those devoted at the moment of this writing. In light of lifestyle changes in new generations, studies analyzing LBP risk factors are crucial. The results can be used in the preventive or educational field, which today represents one of the most effective therapeutic approaches in LBP treatment to avoid pain chronicity and the subsequent economic consequences^8,15^.

The results support the evidence that non-specific LBP is common in adolescence^5^. Indeed, 55.61% (3326 subjects) of students reported having suffered from back pain at least once in their life and 42.62% (2549 subjects) reported one or more episodes of LBP.

LBP lifetime prevalence is a suggestive measure in adolescents: they are more likely to remember pain episodes that occurred also many years earlier, probably because of their emotional, psychological and relational life impact^26^.

In agreement with previous studies, the highest prevalence of LBP was found in the female sex, probably due to a different pain threshold and pain symptom perception^10,27^. Other possible related factors are the greater flexibility of the spine compared to males and the possible changes induced by hormonal changes on the appearance and perception of pain^17,28^.

We did not observe association between LBP and BMI. This is in agreement with previous reports suggesting that non-specific LBP in adolescents is more related to an incorrect lifestyle^5, 8, 21^. Conversely, it has been demonstrated that in adults the risk of low LBP increases in parallel with BMI and may be modulated by physical activity^29^.

We did not identify a clear-cut relationship between physical activity levels (investigated through IPAQ) and LBP; in fact, it emerged that students who regularly practice sports (at least 2-3 hours a week) were less likely to suffer of LBP. These data confirm that physical activity, improving muscle elasticity, strength and likely increasing pain threshold, can prevent the onset of LBP^39,31^.

The relationship between physical activity levels and LBP is controversial and widely discussed. In fact, it has been observed that both an insufficient as well as an excessive motor activity predisposes to the development of LBP with a U-shaped relationship^32^. Specific skills required in different sports expose the vertebral discs to considerable pressures; sports in general increase the risk of injuries which may lead to LBP^33^.

We found an association between LBP and hours of sleep. We observed that sleeping more than five hours a night was associated with a lower probability of suffering from LBP, suggesting that sleep may be a protective factor.

We also found an association between LBP and use of electronic devices (laptop, or tablet or a smartphone) and hours spent sitting^34^, suggesting that the two factors may be related. An inadequate prolonged static posture, adopted using these devices, might generate musculoskeletal overload, activating pain receptors^34^.

Our results confirm a predisposition to LBP in subjects with positive family history, likely due to the genetic factors involved^21, 22, 28, 35^. However, the family environment may also play a role, since it has been observed that parents may impact on pain threshold level and symptoms complain, heightening prevalence of disabling LBP in this sub-sample^21, 22, 28, 35^. Apprehension and anxiety by one or both parents can prompt health-seeking behavior, especially when pain presents a chronic course^36^.

Of 2549 students with LBP, 723 (28.36%) reported at least one episode of non-specific disabling LBP. However, age and gender did not seem to influence the type of LBP (disabling or not).

An association between disabling LBP and hours of sleep was found. Although sleep may be a protective factor for LBP, it has also been reported sufferers of disabling LBP have a poor sleep quality, negatively affecting both the perception of pain and the quality of life^37^.

The presence of a disabling pain increased also the seek for health-care consultation (55.19%). These data are in contrast with previous studies reporting that only 2-15% of children and adolescents with episodes of LBP require a medical and/or instrumental evaluation^11,28^. This difference could be due to the definition of LBP that we use. Most included studies used structured or semi-structured questionnaires with only a partial definition of non-specific LBP, hence it could be misleading to draw comparisons with other studies. In fact, according to our definition, the LBP was non-specific and had to limit the adolescent's daily activities.

Of note, our study recruited more than 5000 adolescents, whereas surveys in this area of research usually consider less than 1500 participants, and the majority of these do not reach 500 individuals^5^. The analysis of such a large sample allows valid and reliable results.

To sum up, these findings stress the need to focus therapeutic efforts with adequate prevention and education programs targeted both to adolescent and relevant adults (e.g., parents, teachers, sports trainers).

*Limitations*

This study has some limitations. The main one is to have used an *ad hoc* questionnaire, which allowed us to obtain data from a large sample, but which is difficult to compare with other studies.

Since different types of schools were included some minimal bias may be introduced in the study, such as the request for longer autonomous hours in equivalent of grammar schools – thus more time spent sitting rather than exercising. Another limitation is that, despite the fact that adolescents can suffer from pain in different segments of the spine, even simultaneously, the formulation of our questionnaire allowed to investigate only one location. Furthermore, the impact of passive and active smoking was not considered. Data on the number and the duration of pain episodes were not included. An investigation of such issues is underway.

Another aspect that we have not considered is the perception of the weight of backpacks by adolescents. It has been reported that, rather than the actual and objective weight of the backpack, it is the student's perception of weight that is associated with LBP^17^.

Another limitation may have been the imperfect recall of events, which is inherently related to this methodological approach and cannot be otherwise corrected.

A potential inclusion bias may have been introduced by the exclusion of subjects with a known diagnosis underlying back pain: although this subgroup does experience LBP, the focus of the present study was LBP not related to spinal diseases. We reckon that we might have included a small sample of individuals in which a diagnosis was not already made, but we are confident that this may not have substantially impacted on final results.

Another limitation to be considered is the incomplete population sample: for convenience, schools were included on an alternating basis (odd zip codes). Although the randomization method is robust, we need to acknowledge that not the whole population aged 14-19 was not included.

Lastly, we did not consider other psychosocial aspects such as depression, anxiety, distress and exposure to stressful life events.

**CONCLUSION**

Our results support the evidence that non-specific LBP is relatively common among adolescents (mostly in females), especially if they are sedentary and heavy users of electronic devices. A positive family history of LBP is associated with disabling LBP, and family environment (apprehension/anxiety/coping skills) might also play a substantial role. Sleeping more than five hours a night is associated with a lower probability of having LBP. Frequently, adolescents with LBP, particularly those with disabling one, consult a healthcare professional. Practicing sport regularly seems to be associated with a lower probability of having LBP. Further studies are needed to identify those at risk and to define more clearly the role of sports activities in this age group, to promote prevention interventions and plan a personalized rehabilitation program.

**Declarations of interest:** none

**Funding:** This work was not supported by grants or funding.

**REFERENCES**

1 Burton AK, Balague F, Cardon G, Eriksen HR, Henrotin Y, Lahad A, et al. European guidelines for prevention in LBP. Eur Spine J 2006;15 Suppl 2:136–68.doi: 10.1007/s00586-006-1070-3.

2 Yuan W, Shen J, Chen L, Wang H, Yu K, Cong H, et al. Differences in non specific low back pain between young adult females with and without lumbar scoliosis. Pain Res Manag 2019;9758273. doi: 10.1155/2019/9758273

3 Ferraro C, Fraschini P, Masiero S, Negrini S, Simonazzi P, Tedeschi C, et al. Trattamento riabilitativo del paziente in eta` evolutiva affetto da patologie del Rachide. In: Rachide e Riabilitazione 2002. Ed. Gruppo di Studio della Scoliosi e delle Patologie Vertebrali, Vigevano (PV), 2003: 5–47.

4 DePalma MJ, Ketchum JM, Saullo T. What is the source of chronic low back pain and does age play a role? Pain Med 2011;12(2):224-33.

5 Calvo-Muñoz I, Kovacs FM, Roqué M, Gago Fernández I, Seco Calvo J. Risk Factors for Low Back Pain in Childhood and Adolescence: A Systematic Review.Clin J Pain 2018;34:468-84. doi: 10.1097/AJP.0000000000000558.

6 Salminen JJ. The adolescent back: a field survey of 370 Finnish school children. Acta Paediatr Scand 1984;315:8–122.

7 Skoffer B, Foldspang A. Physical activity and low-back pain in schoolchildren. Eur Spine J 2008;17:373-9. doi: 10.1007/s00586-007-0583-8.

8 Kovacs FM, Gestoso M, Gil del Real MT, López J, Mufraggi N, Méndez JI. Risk factors for non-specific low back pain in schoolchildren and their parents: apopulation based study. Pain 2003;103:259-68.doi: 10.1016/s0304-3959(02)00454-2.

9 Angarita-Fonseca A, Boneth-Collante M, Ariza-Garcia CL, Parra-Patiño J, Corredor-Vargas JD, Villamizar-Niño AP. Factors associated with non-specific low back pain in children aged 10-12 from Bucaramanga, Colombia: A cross-sectionalstudy.J Back Musculoskelet Rehabil 2019;32:739-47. doi: 10.3233/BMR-160561.

10 Minghelli B, Oliveira R, Nunes C. Non-specific low back pain in adolescents from the south of Portugal: prevalence and associated factors.J Orthop Sci 2014;19:883-92. doi: 10.1007/s00776-014-0626-z.

11 Burton AK, Clarke RD, McClune TD, Tillotson KM. The natural history of LBP in adolescents. Spine 1996;21:2323–8. doi: 10.1097/00007632-199610150-00004.

12 Cakmak A, Yücel B, Ozyalçn SN, Bayraktar B, Ural HI, Duruöz MT, et al. The frequency and associated factors of low back pain among a younger population in Turkey.Spine (Phila Pa 1976) 2004;29:1567-72. doi: 10.1097/01.brs.0000131432.72531.96.

13 Hashem LE, Roffey DM, Alfasi AM, Papineau GD, Wai DC, Phan P, et al. Exploration of the inter-relationships between obesity, physical inactivity, inflammation, and low back pain. Spine (Phila Pa 1976) 2018;43:1218-24.doi: 10.1097/BRS.0000000000002582.

14 Sribastav SS, Long J, He P, He W, Ye F, Li Z, et al. Risk factors associated with pain severity in patients with non-specific low back pain in southern china. Asian Spine J 2018;12:533-43. doi: 10.4184/asj.2018.12.3.533.

15 Harreby M, Neergaard K, Hesselsoe G, Kjer J. Are radiologic changes in the thoracic and lumbar spine of adolescents risk factors for LBP in adults? A 25-year prospective cohort study of 640 school children. Spine 1995;20:2298–302. doi: 10.1097/00007632-199511000-00007.

16 Zadro JR, Shirley D, Duncan GE, Ferreira PH. Familial factors predicting recovery and maintenance of physical activity in people with low back pain: Insights from a population-based twin study. Eur J Pain 2019;23:367-77.doi: 10.1002/ejp.1311.

17 Negrini S, Carabalona R. Backpacks on schoolchildren’s perceptions of load, associations with back pain and factors determining the load. Spine 2002;27:187–95. doi: 10.1097/00007632-200201150-00014.

18 Moroder P, Runer A, Resch H, Tauber M.Low back pain among medical students. Acta Orthop Belg 2011;77:88-92.

19 Deyo RA, Weinstein JN.Low back pain. N Engl J Med 2001;344:363-70.doi: 10.1056/NEJM200102013440508.

20 Heneweer H, Vanhees L, Picavet HS. Physical activity and low back pain: a U-shaped relation?Pain 2009;143:21-5. doi: 10.1016/j.pain.2008.12.033.

21 Masiero S, Carraro E, Celia A, Sarto D, Ermani M. Prevalence of nonspecific low back pain in schoolchildren aged between 13 and 15 years. Acta Paediatr 2008;97:212-6. doi: 10.1111/j.1651-2227.2007.00603.x.

22 Masiero S, Carraro E, Sarto D, Bonaldo L, Ferraro C. Healthcare service use in adolescents with non-specific musculoskeletal pain. Acta Paediatr 2010;99:1224-8.doi: 10.1111/j.1651-2227.2010.01770.x.

23 Craig CL, Marshall AL, Sjöström M, Bauman AE, Booth ML, Ainsworth BE,et al. International physical activity questionnaire: 12-Country reliability and validity. Med Sci Sports Exerc 2003;35:1381-95. doi: 10.1249/01.MSS.0000078924.61453.FB.

24 von Elm E1, Altman DG, Egger M, Pocock SJ, Gøtzsche PC, Vandenbroucke JP; STROBE Initiative. The Strengthening the Reporting of Observational Studies in Epidemiology (STROBE) statement: guidelines for reporting observational studies.J Clin Epidemiol 2008;61:344-9. doi: 10.1016/j.jclinepi.2007.11.008.

25 R Core Team (2019). R: A language and environment for statistical computing. R Foundation for Statistical Computing, Vienna, Austria. URL https://www.R-project.org/

26 Jaaniste T, Noel M, von Baeyer CL.Young children's ability to report on past, future, and hypothetical pain states: a cognitive-developmental perspective.Pain 2016;157:2399-409. DOI: 10.1097/j.pain.0000000000000666.

27 Keogh E, Eccleston C.Sex differences in adolescent chronic pain and pain-related coping.

Pain 2006;123:275-84. doi: 10.1016/j.pain.2006.03.004.

28 Balagué F, Damidot P, Nordin M, Parnianpour M, Waldburger M. Cross-sectionalstudy of the isokinetic muscle trunk strength among school children. Spine (PhilaPa 1976) 1993;18:1199-205. doi: 10.1097/00007632-199307000-00013.

29 Smuck M, Kao MC, Brar N, Martinez-Ith A, Choi J, Tomkins-Lane CC. Does physical activity influence the relationship between low back pain and obesity? Spine J 2014;14:209-16.

30 Roth-Isigkeit A, Thyen U, Stöven H, Schwarzenberger J, Schmucker P. Pain amongchildren and adolescents: restrictions in daily living and triggering factors.Pediatrics 2005;115:e152-62. doi: 10.1542/peds.2004-0682.

31 Muntaner-Mas A, Palou P, Ortega FB, Vidal-Conti J. Sports participation andlow back pain in schoolchildren. J Back MusculoskeletRehabil 2018;31:811-9. doi: 10.3233/BMR-171062.

32 Sjolie AN. Associations between activities and low back pain in adolescents. Scand J Med Sci Sports 2004;14:352-9. doi: 10.1111/j.1600-0838.2004.377.x.

33 Trompeter K, Fett D, Platen P. Prevalence of Back Pain in Sports: A Systematic Review of the Literature. Sports Med 2017;47:1183-1207. doi: 10.1007/s40279-016-0645-3.

34 Bento TPF, Cornelio GP, Perrucini PO, Simeão SFAP, de Conti MHS, de Vitta A. Low back pain in adolescents and association with sociodemographic factors, electronic devices, physical activity and mental health.J Pediatr (Rio J) 2019;pii:S0021-7557(19)30279-7. doi: 10.1016/j.jped.2019.07.008.

35 Galozzi P, Maghini I, Bakdounes L, Ferlito E, Lazzari V, Ermani M,et al. Prevalence of low back pain and its effect on health-related quality of life in 409 scholar adolescents from the Veneto region. Reumatismo 2019;71:132-40. doi: 10.1016/j.jped.2019.07.008.

36 Eccleston C, Crombez G, Scotford A, Clinch J, Connell H. Adolescent chronic pain: patterns and predictors of emotional distress in adolescents with chronic pain and their parents. Pain 2004;108:221–9. doi: 10.1016/j.pain.2003.11.008.

37 You DS, Albu S, Lisenbardt H, Meagher MW. Cumulative childhood adversity as a risk factor for common chronic pain conditions in young adults. Pain Med 2019;20:486-94. doi: 10.1093/pm/pny106.

**FIGURE LEGENDS**

**Figure 1.** Distribution of disabling (dis) low back pain (LBP) or non-dis LBP and A) maximum pain intensity and B) mean pain intensity.

**Supplementary figure 1.** Flow chart showing the inclusion process of participants. Response rate 100%.

**Supplementary figure 2.** Distribution of sex and age (in years) among interviewed students.
