# Supplementary figures and images for: Lifetime Prevalence of Nonspecific Low Back Pain in Adolescents: A Cross-sectional Epidemiologic Survey
Source: Am J Phys Med Rehabil. 2021 Feb 19;100(12):1170–5. doi: 10.1097/PHM.0000000000001720 (PMC9988216; doi:10.1097/PHM.0000000000001720)

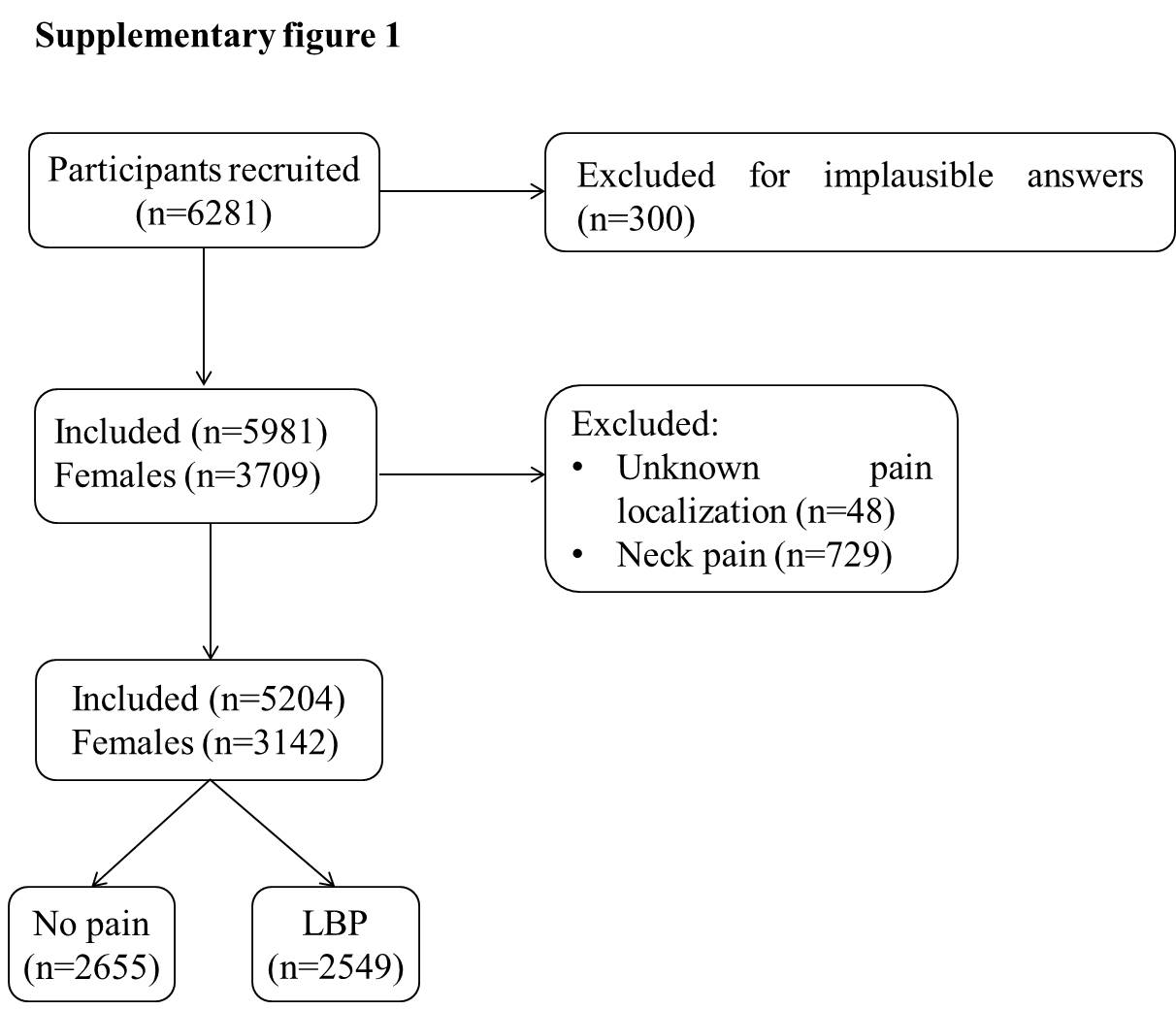

Supplement: Supplementary file 2 [file ajpmr-100-1170-s002.tif]

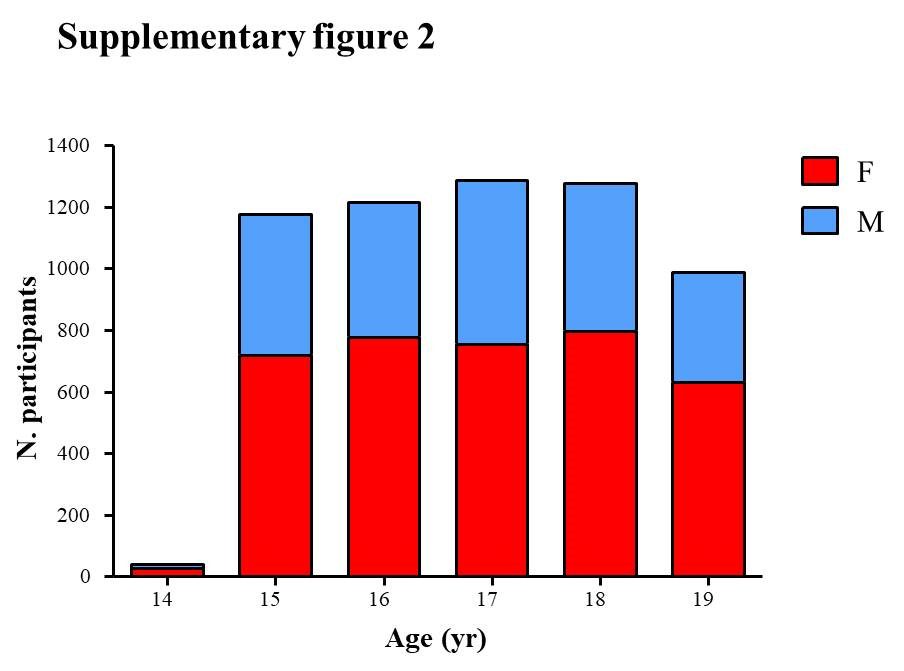

Supplement: Supplementary file 3 [file ajpmr-100-1170-s003.tif]
